# Pancreatic cancer and depression: myth and truth

**DOI:** 10.1186/1471-2407-10-569

**Published:** 2010-10-20

**Authors:** Martina Mayr, Roland M Schmid

**Affiliations:** 1 Internal Medicine II, Klinikum Rechts der Isar, Technical University of Munich, Germany

## Abstract

**Background:**

Various studies reported remarkable high incidence rates of depression in cancer patients compared with the general population. Pancreatic cancer is still one of the malignancies with the worst prognosis and therefore it seems quite logical that it is one of the malignancies with the highest incidence rates of major depression.

However, what about the scientific background of this relationship? Is depression in patients suffering from pancreatic cancer just due to the confrontation with a life threatening disease and its somatic symptoms or is depression in this particular group of patients a feature of pancreatic cancer per se?

**Discussion:**

Several studies provide evidence of depression to precede the diagnosis of pancreatic cancer and some studies even blame it for its detrimental influence on survival. The immense impact of emotional distress on quality of life of cancer patients enhances the need for its early diagnosis and adequate treatment. Knowledge about underlying pathophysiological mechanisms is required to provide the optimal therapy.

**Summary:**

A review of the literature on this issue should reveal which are the facts and what is myth.

## Background

Various studies have demonstrated the immense impact of psychological distress on life quality and illness trajectory in pancreatic cancer patients. Moreover, Bultz et al. entitled emotional distress "the sixth vital sign in cancer care" [1,2]. Accordingly they requested health care providers to attach no less importance to the monitoring of emotional distress than to the monitoring of "traditional" vital signs such as blood pressure or heart rate. Patients may benefit from early recognition and adequate treatment of emotional burden or even depression as documented in several studies[3]. However it is still a matter of debate whether individual psychological coping strategies have significant impact on overall survival in cancer patients [4].

Pancreatic cancer in particular is one of the tumor entities with a strikingly high incidence of depression as well as one of those with the worst prognosis. Is this just a coincidence or a sign of a reciprocal interaction? What is the impact of psyche on cancer mortality? To address this issue we reviewed published data on pancreatic cancer related depression for evidence of a relationship between psychological distress and the course of this disease.

## Discussion

### Pancreatic cancer and depression

A comprehensive study by Hinz et al. has reported that the prevalence of anxiety and depression in cancer patients is nearly as twice as high as in the general population [5]. This observation is consistent with data from the large nord-trondelag health study by Stordal et al. [6]. Several preceding studies reported similar results but a few did not show elevated depression scores related to cancer [7-10]. Regarding the diversity of studies on this topic any inconsistency in findings can be mainly attributed to low sample size, the lack of differentiation by gender, age and time since diagnosis, treatment and other methodological flaws as well [5,10-12]. Those studies evaluating different tumor entities separately have remarkably high depression rates associated with specific tumor types in common. In particular patients with pancreatic, lung, oropharyngeal and breast cancer are seriously affected by emotional burden and major depression [10,11,13,14]. In this respect pancreatic cancer is the tumor entity with the highest incidence rate of depression among all other tumors of the digestive system [15-17].

This strikingly close relationship between pancreatic cancer and major depression has been known for more than 70 years [18]. The initial description dates back to Yaskin in 1931 [19]. His report on the association between pancreatic cancer and the triad of anxiety, depression and "sense of impending doom" was the origin of several ensuing studies [18-20]. In 1967 Fras et al. reviewed the available "original contributions, which mentioned mental symptoms as a part of symptomatology of carcinoma of the pancreas". In this study depression was diagnosed in 76% of the patients with pancreatic cancer and only in 20% of the patients with colon cancer. This investigation was made prior to surgery and more than 50% of the patients with pancreatic cancer reported psychological symptoms, up to 43 months before somatic complaints [15]. This aroused a debate about "mental symptoms as an aid for early diagnosis of pancreatic cancer" [21]. Jacobsson et al. found initial depression in 14% of patients who were afterwards diagnosed with cancer of the pancreas but only in 4% of patients with subsequent diagnosis of gastric cancer [22]. A similar study by Joffe et al. 1986 reported severe depression in over half the patients who were finally diagnosed with pancreatic cancer but none in the patients with gastric cancer [23]. Some case reports inscribe psychological symptoms like panic or anxiety as a harbinger of the subsequent tumor diagnosis [24,25]. Unexpected high incidence rates were confirmed by a study in 1993 showing symptoms of depression in 71% of the examined pancreatic cancer patients and "anxiety-related symptoms" in 48% of the patients [26]. In the aforementioned comprehensive review by Massie et al. the prevalence of depression in pancreatic cancer was between 33 and 50% [10].

For most of the studies have investigated patients recently diagnosed with cancer, the possibility of recall bias has be to be considered. Holland et al. employed self-assessment questionnaires to assess depression, anxiety, fatigue and mood swings in patients with advanced cancer [16]. Finally patients with pancreatic cancer had higher self-ratings of depression than patients with other advanced abdominal tumors. In contrast a prospective study by Labori et al. which employed self-report measures as the Edmonton Symptom Assessment Scale (ESAS) also noted relatively low levels of depression in patients with advanced pancreatic carcinoma, but the small sample size of this study and the large standard deviations put the definite impact of the result up for discussion [27,28]. These methodological limitations complicate the final appraisal of the available studies. Another challenge is the differentiation between major depression and the "quite normal" psychological distress of patients coping with cancer diagnosis. So there is indeed a demand for an appropriate instrument to identify cancer related, cancer specific depression.

### Diagnosis of depression-screening tools

The reliability of any data on the incidence of depression in cancer patients is highly depended on the appropriate choice of the assessment tool. The respective tool has to be suitable not only for differentiation between depression and "quite normal" psychological distress of patients coping with cancer diagnosis but also for avoiding misattribution of symptoms of the tumor disease to depression [20]. Unfortunately studies on the predictive value of the different screening tools are sparse [29,30]. The basic structures of the established depression assessment tools are mainly diagnostic interviews, patient self-report forms or observer ratings [31]. In general, the classification of depression as a variety of affective disorders complies with the Diagnostic and Statistical Manual of Mental Disorders DSM-IV edited 1994 by American Psychiatric Association (corresponding to ICD-10). The current version DSM-IV-TR was published July 2000, a revised version, the DSM-V is being developed at present.

The anxiety disorders interview schedule for DSM-IV, a structured clinical interview, may be employed to assess a range of disorders going through different items [29,32]. The DSM-IV-classification was often questioned to be adequate for application in cancer patients. The advantage of structured clinical interviews is undoubted, but they have to be conducted by skilled staff and therefore require enormous amounts of resources [31]. Patient self-report screening inventories are brief enough and therefore more useful in routine application in cancer patients [30]. Furthermore, self-report forms are supposed to be more sensitive to identify significant functional impairment during follow up [29]. Therefore, it is often recommended to use self-report forms as a first step to identify depression followed by a structured interview in the emerging cases [31]. Up to the present time a variety of assessment instruments have been developed and the different forms were employed in multiple studies. The most common ones applied in cancer patients are referred to below.

The **Hamilton Rating Scale for Depression **(**HAMD**) has been a standard instrument for peer assessment of depression's severity since 1970 [33]. Lin et al. most recently used the HAMD-24 as the rating scale for depression to screen patients with gastrointestinal tumors [17]. HAMD-24 contains 24 items which are recalled within the modified HAMD version of 1985 [34]. But HAMD as well as **CPRS **(Comprehensive Psychopathological Rating Scale) or **MADRS **(Montgomery-Asberg Depression Rating Scale) are in fact no adequate diagnostic instruments for depression but measures of illness severity and it is still debatable whether these rating scales are suitable for primary identification of major depression [11,35-37]. In cancer patients diagnosis of depression requires special effort because somatic symptoms of depression like fatigue, anorexia and weight loss are quite common in malignancies. Therefore the focus has to be on evaluation of psychological and cognitive symptoms like hopelessness, loss of ambition, anhedonia or suicidal ideation [18,38,39]. To meet these demands assessment instruments like the **Distress Thermometer, **the **Brief Symptom Inventory **(**BSI**), the **Symptom Checklist 90-R **(**SCL-90**) or the **Problem Common Checklist **have been designed [9,14,28,40]. The currently most established self-assessment instruments are the **Becks Depression Inventory **(**BDI**) and the **Hospital Anxiety and Depression Scale (HADS) questionnaire**. The BDI has been developed for clinical implementation of psychiatric patients to rate severity of depression symptoms and may be combined with the Beck Hopelessness Scale (BHS) to assess cognitive aspects of depression [41]. But it comprises some somatic items which may cause false positive results by the influence of underlying disease and concomitant therapy [31]. The Hospital and Anxiety Depression Scale (HADS) has been designed to avoid in particular an immoderate influence of somatic symptoms in screening for depression [11]. Its validity and reliability in cancer patients has been demonstrated in several studies [31].

### Pathophysiological aspects of cancer related depression

There are two pivotal questions to address. First, is depression a specific feature of cancer or could it be traced to the disease's severe concomitant symptoms and its fatal prognosis? - Second, is depression itself a promoter of tumor development or are pathophysiological changes due to cancer responsible for the high incidence of depression?

One possible approach to the evident bidirectional relationship between depression and pancreatic cancer is to regard the extent of psychological distress patients experience being faced with such fatal diagnosis. For pancreatic cancer patients the impact of medical, psychological and social factors is generally more severe than for patients with other intestinal cancers [18]. Another approach to the origin of cancer-related depression comprises the research of possible pathophysiological mechanisms.

Some authors ascribe cancer related depression to paraneoplastic limbic encephalitis [42,43] - but to confirm this diagnosis a range of neuropathological tests are necessary. Symptoms of paraneoplastic limbic encephalitis often precede cancer diagnosis as depression precedes pancreatic cancer [20]. Almost 50% of pancreatic cancer patients show depressive symptoms in advance of their tumor diagnosis [18,20,26]. In a study with strikingly high incidence of depression in pancreatic cancer Lin et al. reported no statistically significant difference in patients knowing diagnosis or not [17]. Among these patients development of depression was independent of the awareness of "impending doom". Accordingly it is still under discussion whether pancreatic cancer induces depression or whether depression enhances the risk of tumor development [20,44].

In this debate a dysregulated immune response is supposed to be the causal link between depression and cancer. Animal models indicate that proinflammatory cytokines can elicit "depression-like" symptoms [12,45]. In pancreatic cancer patients indeed increased levels of several cytokines such as IL-6, IL-18 and TNF-alpha can be found. These factors may have impact on the hypothalamic-pituitary-adrenal (HPA) axis and the CRF (corticotropin-releasing factor). Some authors assume this pathway to be the cause for pathologic results in the dexamethason suppression test, in analogy to a paraneoplastic symptom. Elevated IL-6 plasma levels in particular seem to be associated with major depression in pancreatic cancer patients and tumor cells can influence production of serotonin receptor antibodies active in central nervous system [23,46-49].

### Antidepressant therapy - an essential part of supportive care

Depression is one of the crucial factors impairing life quality of cancer patients. Accordingly early and adequate antidepressant treatment is an essential component of all-embracing supportive care. Management of depression requires sufficient pain control and psychological support as well as use of antidepressants [50]. Any possible organic causes of depression like metabolic disorder, infections, drug side effects (i.e. chemotherapeutic substances) or brain radiation should be considered before administration of pharmacologic therapy [18,39].

Clinical trials have shown the efficacy of various antidepressants to be comparable [18,39,50]. The choice of the appropriate antidepressant substance should be determined by the main target symptoms referring to the patient's current medical problems and the side effect profile [50]. Among the variety of antidepressants and antipsychotic drugs a selection of the most established ones is listed below.

Tricyclic antidepressants are the most commonly administered drugs [11,18,51]. They may even be effective as concomitant therapy for patients with neuropathic pain, insomnia and specific cholinergic symptoms. There is an unexplained smaller therapeutic range of tricyclic agents in cancer patients which is to be considered [18]. Due to increased serotonin levels in pancreatic cancer selective serotonin re-uptake inhibitors (SSRIs) may be effective as a kind of "targeted therapy" [20]. Mirtazapine, a specific serotonergic antidepressant, demonstrated higher sustained remission rate than amitriptylin and more rapid onset in several studies. Therefore mirtazapine is applicable even in elderly patients and recommended for those with a short life expectancy. Besides it is assumed to have effects on pain, nausea, emesis and appetite loss [52-54]. Amisulpride as an atypical antipsychotic enhances dopaminergic transmission in the frontal cortex and limbic areas. Concomitant treatment during chemotherapy achieved significant reduction of depressive symptoms with minimal side effects [55]. Patients with prevalent symptoms of anxiety receive better treatment response with benzodiazepines [18,38,48]. But benzodiazepines may increase psychomotoric slowing and fatigue which is a very general complaint of cancer patients. Hence different drugs have been tested to decrease weakness. Among various psychostimulants methylphenidate is the most established, but firstly the results of the different trials are inconsistent and secondly tolerance may develop [18,20,39]. Finally some authors refer to current guidelines which suggest psychostimulants "to be reasonable to consider for severe fatigue" [56].

Beyond pharmacologic treatment of depression supportive psychotherapy including teaching coping strategies plays an important role in handling cancer related depression. Even if it is regarded as an essential treatment approach restricted life expectancy has to be taken in account when constituting the type of psychotherapy or supportive counseling [11,18,39].

### Depression and cancer mortality

The relationship between affective disorders and mortality has been in the scientific focus for more than 60 years. Kerr et al. reported 1969 an "increased mortality rate from physical disease in patients with affective disorders, particularly from carcinoma" [57]. They also noticed remarkably high rates of pancreatic cancer and lung carcinoma among these patients. The following decades numerous investigations on depression in cancer patients have been conducted. High prevalence rates of depression particularly in patients suffering from tumor entities with worse prognosis like pancreatic cancer led to the crucial question for the impact of depression on mortality. The history of depression in cancer patients has been linked to worse overall survival rates due to impaired immune response and higher rates of suicide in some but not all studies [11,38,58]. In a retrospective analysis of survival data of 258 patients by Sheibani-Rad et al. pancreatic cancer associated depression did not affect survival [59].

Vice versa the effect of antidepressant therapies on mortality was investigated. First descriptions of the influence of psychotherapeutic interventions on the survival of tumor patients were published over 30 years ago. Some studies reported that group therapies led to prolonged survival rates in the participating patients [60]. They were sharply criticized for methodical flaws and bias like the poor survival data of the control group but the question for the linkage between psychological factors and the course of cancer remained.

Several reviews and meta-analyses examined the supposed linkage critically. In 2002 Petticrew et al. reviewed a selection of prospective observational studies to judge the impact of psychological coping styles on the survival from or the recurrence of cancer. Consistent evidence for the often cited bilateral relationship could hardly be asserted. The authors stated the positive results to be "confined to small and methodologically flawed studies". In agreement with other authors they assumed influential publication bias [61].

Nevertheless more and more trials on this topic have been conducted in recent years. Almost all report on the high incidence of depressive symptoms and consequently, the significant impact on survival. In 2007 Steel et. al performed a noteworthy prospective study to establish a meditational model of depression, immunity and survival in patients with hepatobiliary carcinoma [58]. As soon as the paper was published a counterstatement put it up for discussion due to a variety of confounding factors including the small sample size [62]. Currently a meta-analysis on depression and cancer mortality by Pinquart et al. reassessed the reliability of results depending on varying study characteristics. Finally "the associations between depression and mortality persisted after controlling for confounding medical variables", but the authors emphasize the demand for further research also [4,61].

## Summary

The phenomenon of cancer related depression has been observed and investigated in every possible way over the last decades. Most of the studies demonstrate strikingly high incidence rates of depression in cancer patients particularly among those with fatal prognosis like pancreatic cancer. Moreover, a recently published trial complains about a lack of attention to psychological distress in pancreatic cancer patients [28] - although the immense impact of depression on quality of life has been established and meanwhile assessment of life quality is highly standardized. Comparing pancreatic cancer patients with and without depression a significant deterioration of life quality has been demonstrated. In clinical studies patients with pancreatic cancer showed most significant impairment in scores of physical, emotional and cognitive functioning with a special impact on social functioning. The score of symptom scales in particular fatigue and pain was significantly higher in the pancreatic cancer group comparing it to other intra-abdominal malignancies [17]. The association between pain and depression is well documented [38,41]. It is still under discussion whether the presence of depression increases the risk of chronic pain per se or suffering from chronic pain evolves major depression itself [11], but chronic pain is undoubtedly a crucial factor in judging quality of life [50].

Concerning the linkage between depression and the course of cancer and survival a lot of studies were conducted. Several of them described a strong relationship between depression and mortality and some could not detect a linkage at all. With regard to comprehensive meta-analyses there is a tendency for a correlation between higher levels of depressive symptoms and elevated mortality, but judgment of available study results is confined to small sample sizes and various confounding factors [4,61]. Investigations concerning the pathophysiological background inspired the hope for a better understanding of the correlation between somatic and psychiatric illness and the possible impact on mortality. Disorders in endocrinological and immunological pathways have been supposed to be responsible for the influence of negative psychological aspects on survival but these biochemical changes have even been assumed to elicit depressive reactions themselves [63,64]. It is a feasible aspect that the correlation between emotional disorders and illness trajectory is confounded by the often poor clinical state of the patients. The poor clinical state itself is associated with a poor prognosis and its somatic symptoms are highly correlated with depressive reactions [65,66]. So the employed assessment tools for depression should minimize the influence of clinical symptoms.

In summary there is no final explanation for the linkage between pancreatic cancer, depression and survival and there is no reliable evidence that psychological coping styles may definitely influence survival or recurrence of cancer [61]. Therefore cancer patients should not be blamed of worsening their situation when not participating in group interventions or psychotherapeutic treatment [62]. But review of the data published so far confirms the utmost importance of paying attention to depressive reactions in cancer patients and care for early adequate treatment because the impact on quality of life is assured and for patients suffering from a malignancy with such a poor prognosis like pancreatic cancer improving quality of life has to be the major therapeutic goal. Actually dealing with this topic the frontiers between reality and myth are merging and further investigation is required to determine the extraordinary correlation of psychological and pathophysiological findings in pancreatic cancer.

## Competing interests

The authors declare that they have no competing interests.

## Authors' contributions

All authors have contributed in writing the review. All the authors read and approved the final manuscript.

## Pre-publication history

The pre-publication history for this paper can be accessed here:

http://www.biomedcentral.com/1471-2407/10/569/prepub
